# Achieving High Thermoelectric Performance in Rare-Earth Element-Free CaMg_2_Bi_2_ with High Carrier Mobility and Ultralow Lattice Thermal Conductivity

**DOI:** 10.34133/2020/5016564

**Published:** 2020-07-24

**Authors:** Muchun Guo, Fengkai Guo, Jianbo Zhu, Li Yin, Qian Zhang, Wei Cai, Jiehe Sui

**Affiliations:** ^1^National Key Laboratory for Precision Hot Processing of Metals, Harbin Institute of Technology, Harbin 150001, China; ^2^Department of Materials Science and Engineering, Harbin Institute of Technology, Shenzhen, Guangdong 518055, China

## Abstract

CaMg_2_Bi_2_-based compounds, a kind of the representative compounds of Zintl phases, have uniquely inherent layered structure and hence are considered to be potential thermoelectric materials. Generally, alloying is a traditional and effective way to reduce the lattice thermal conductivity through the mass and strain field fluctuation between host and guest atoms. The cation sites have very few contributions to the band structure around the fermi level; thus, cation substitution may have negligible influence on the electric transport properties. What is more, widespread application of thermoelectric materials not only desires high *ZT* value but also calls for low-cost and environmentally benign constituent elements. Here, Ba substitution on cation site achieves a sharp reduction in lattice thermal conductivity through enhanced point defects scattering without the obvious sacrifice of high carrier mobility, and thus improves thermoelectric properties. Then, by combining further enhanced phonon scattering caused by isoelectronic substitution of Zn on the Mg site, an extraordinarily low lattice thermal conductivity of 0.51 W m^−1^ K^−1^ at 873 K is achieved in (Ca_0.75_Ba_0.25_)_0.995_Na_0.005_Mg_1.95_Zn_0.05_Bi_1.98_ alloy, approaching the amorphous limit. Such maintenance of high mobility and realization of ultralow lattice thermal conductivity synergistically result in broadly improvement of the quality factor *β*. Finally, a maximum *ZT* of 1.25 at 873 K and the corresponding *ZT*_*ave*_ up to 0.85 from 300 K to 873 K have been obtained for the same composition, meanwhile possessing temperature independent compatibility factor. To our knowledge, the current *ZT*_*ave*_ exceeds all the reported values in AMg_2_Bi_2_-based compounds so far. Furthermore, the low-cost and environment-friendly characteristic plus excellent thermoelectric performance also make the present Zintl phase CaMg_2_Bi_2_ more competitive in practical application.

## 1. Introduction

The development of clean energy could help to alleviate the energy crisis [1, 2]. Recently, solid-state thermoelectric (TE) technology has received a great deal of attention because of its ability to directly convert the waste heat into desirable electricity [3]. The conversion efficiency of thermoelectric device is determined by the dimensionless figure-of-merit *ZT* = *S*^2^*σT*/(*κ*_E_ + *κ*_L_), in which *S*, *T*, *σ*, *κ*_*E*_, and *κ*_*L*_ are Seebeck coefficient, absolute temperature, electrical conductivity, electronic thermal conductivity, and lattice thermal conductivity, respectively [4]. However, the intertwined correlation among the *S*, *σ*, and *κ*_*E*_ hinders the further enhancement of *ZT* value. Thus, some concepts and strategies including band convergence [5, 6], band nestification [7], resonant level [8, 9], and energy filtering [10, 11] have been successfully adopted to optimize the electrical transport properties. On the other hand, progresses on reducing the lattice thermal conductivity *κ*_*L*_, the sole independent parameter, are also achieved by introducing nanostructuring [12, 13], point defect [14–16], dislocation [17, 18], lattice anharmonicity [19, 20], as well as liquid-like phonons [21, 22] or exploring materials with complex crystal structure [23–25].

Zintl phases seem congenital thermoelectric materials for their structural complexity and inherently low thermal conductivity [26, 27]. Some representative compounds, including Yb_14_MnSb_11_ [28], Ca_1-x_Yb_x_Zn_2_Sb_2_ [29], Mg_3_Sb_2_ [30], *β*-Zn_4_Sb_3_ [31], Ca_9_Zn_4_Sb_9_ [32], and (Eu_0.5_Yb_0.5_)_1-x_Ca_x_Mg_2_Bi_2_ [33], have been exploited for their prominent thermoelectric performance. Particularly, the p-type AM_2_X_2_ (1-2-2 type) compounds with CaAl_2_Si_2_-type structure deserve intensive attention because of their higher carrier mobility originated from unique crystal structure, where the covalently bonded anionic sheets (M_2_X_2_)^2-^ sandwiched between A^2+^ cations planes (A contains alkaline earth or divalent rare earth element, M is transition metal or main-group element, X comes from element Sb or Bi) [34]. As one of the two major categories of 1-2-2 type materials, Sb-based system has been paid lots of attention and is in-depth studied. However, it is a pity that the toxic element Cd is always inevitable among Sb-based materials with maximal *ZT* in excess of 1 [35–39], which is not in keeping with the concept of environmental friendliness. Out of the environmental consideration, peoples gradually turn their concerns to the analogous Bi-based Zintl materials, AMg_2_Bi_2_ (A = Ca, Yb, Eu) [40, 41].

In the Bi-based family, CaMg_2_Bi_2_ compound possesses the virtues of environmental friendliness, low cost, and high element abundance compared with the other two containing expensive Yb and Eu. Early studies reported that the peak *ZT* is only ~0.1 for CaMg_2_Bi_2_ synthesized by the traditional melting method, which is far from practical application [41]. Shuai et al. utilized the ball milling method to reduce the element volatilization and then selected the monovalence Na^+^ to replace Ca^2+^, which eventually causes a mediocre *ZT* ≈ 0.9 at 873 K for Ca_0.995_Na_0.005_Mg_2_Bi_1.98_ sample [42]. However, the trace alloying fractions of alkali-metal and weak mass/size difference between alkali-metal and Ca have a faint influence on the lattice thermal conductivity [42]. Attempts at introducing point defect and utilizing multiscale phonon engineering in CaMg_2_Bi_2_ have been executed to further reduce the *κ*_*L*_ [33, 43]. Even though the peak *ZT* value as high as ~1.3 at 873 K in Ca_0.6_Yb_0.2_Eu_0.2_Mg_2_Bi_2_ is obtained, the expensiveness of some rare-earth elements makes it difficult to achieve large scale application in some cases [33].

In other doped CaMg_2_Bi_2_ systems, the element Ba has been verified to be as effective as Yb or Eu in reducing *κ*_*L*_ [44]. Further considering that Bi-*p* orbitals dominate the valence band of CaMg_2_Bi_2_ [33], element doping on Ca site may have a minor effect on valence band structure, which is benefited for maintaining high carrier mobility. Thus, starting with the adjusted composition, Ca_0.995_Na_0.005_Mg_2_Bi_1.98_ [42], the substitution of isoelectronic Ba for Ca will not only tailors carrier concentration while maintaining the high carrier mobility but also reduces the *κ*_*L*_ by constructing point defects, leading to an enhanced thermoelectric properties. Subsequently, Zn doping on Mg site is carried out to further reduce the *κ*_*L*_. This equivalent substitution can effectively block phonon transmission without significantly disordering the electrical property. Eventually, the maximum *ZT* value of ~1.25 at 873 K and the average *ZT* (*ZT*_*ave*_) of ~0.85 between 300 K and 873 K are gained in (Ca_0.75_Ba_0.25_)_0.995_Na_0.005_Mg_1.95_Zn_0.05_Bi_1.98_ sample. As well, this *ZT*_*ave*_ is the highest value in p-type AM_2_X_2_ Zintl compounds ever reported, and the richness and nontoxic constituent elements are of great practical significance to promote the application of large-scale and high-efficiency thermoelectric modules.

## 2. Result and Discussion

### 2.1. The Effect of Ba Doping on Enhancing TE Properties

**Figure 1:**
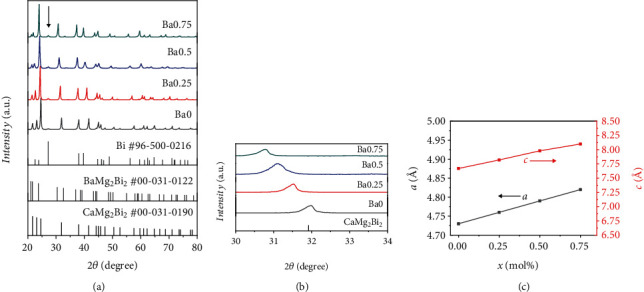
(a) The room-temperature XRD patterns for Ba*x* (*x* = 0, 0.25, 0.5, 0.75) alloys. (b) The zoomed-in XRD patterns between 30° and 34°. (c) Ba concentration-dependent lattice parameters.

The details of sample preparation and characterization could be found in supporting information.  Figure 1 shows the XRD patterns and the related lattice parameters of Ba*x* (*x* = 0, 0.25, 0.5, 0.75) alloys. The diffraction peaks can be nicely indexed to trigonal CaAl_2_Si_2_ structure with the space group *P*$\bar{3}$*m*1. There is minor impurity phase Bi existing between 25° and 30° on account of the loss of Ca and Mg at elevated temperature. However, it should be noted that the Bragg peaks shift toward lower diffraction angles with increasing Ba fraction as shown in Figure 1(b), indicating an enlarged lattice parameter derived from the larger ionic radius of Ba^2+^ (1.35 Å) compared to that of Ca^2+^ (1.0 Å). As anticipated, the calculated lattice parameters *a* and *c* linearly increase with increasing Ba content as shown Figure 1(c).

**Figure 2:**
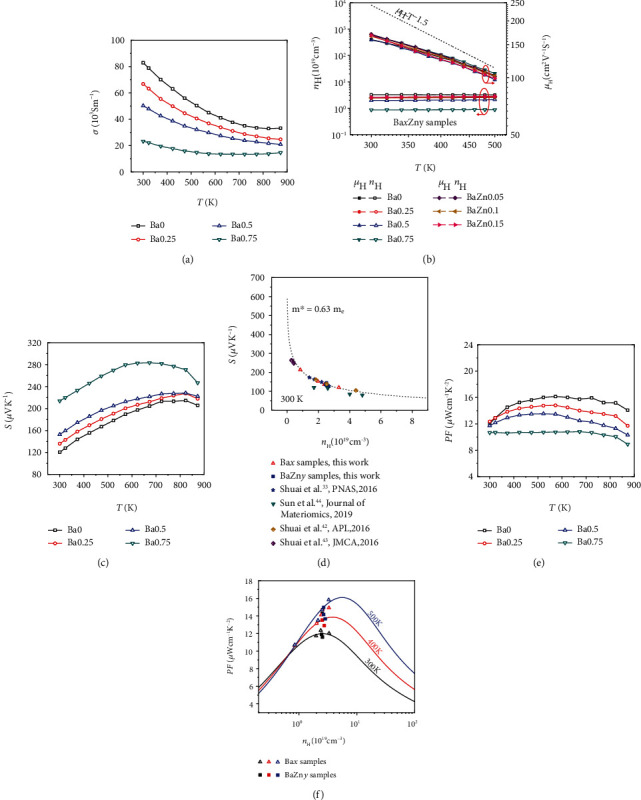
(a) The electrical conductivity as a function of temperature for Ba*x* (*x* = 0, 0.25, 0.5,0.75) samples. (b) Temperature-dependent carrier concentration and mobility for Ba*x*Zn*y* (*x* = 0, 0.25, 0.5, 0.75; *y* = 0, 0.05, 0.1, 0.15). The dotted line represents the relationship of *μ*_*H*_ ~T^−1.5^. (c) Temperature-dependent Seebeck coefficient for Ba*x* samples. (d) Room temperature Seebeck coefficient *vs* carrier concentration for our work and the previously reported data [33, 42–44], where the black curve is calculated Pisarenko plot with *m*^∗^ = 0.63 *m*_*e*_. (e) Variation of the *PF* for Ba*x* samples. (f) Carrier concentration dependent *PF* at different temperatures. The solid lines are calculated based on SPB with the hypothesis of the insensitive *μ*_*H*_ to carrier concentration. The fitting *μ*_*H*_ values are 164, 130, and 102 cm^−2^ V^−1^ s^−1^ at 300 K, 400 K, and 500 K, respectively.

Figure 2 depicts the electrical transport properties of Ba*x* (*x* = 0, 0.25, 0.5, 0.75) alloys. The decreasing trend of *σ* as temperature increasing represents a typical degenerate semiconducting behavior. Moreover, the electrical conductivity decreases with increasing Ba content during the whole measured temperature range, which could be attributed to the decrease of carrier concentration since the mobility changes slightly with the doping fraction grows as shown in Figure 2(b) and Table S1.

To further disclose the electrical transport mechanism after doping, the *n*_*H*_ and *μ*_*H*_ as a function of temperature for Ba*x*Zn*y* alloys (*x* = 0, 0.25, 0.5, 0.75; *y* = 0, 0.05, 0.1, 0.15) are shown in Figure 2(b). Although Ba and Ca have the same valence state in the Zintl phase, the difference of electronegativity might give rise to diverse electrical properties. The electron transfer may become more distinct in Ba-doped sample because of the higher electropositive of Ba than Ca, which eventually leads to the lower *n*_*H*_ of Ba-doped samples. The similar phenomenon also appears in Ca_1-x_Yb_x_Mg_2_Bi_2_ [43] and Yb_1-x_Ca_x_Zn_2_Sb_2_ materials [45]. Thus, utilizing the difference of electronegativity does effectively tailor the *n*_*H*_, which is a crucial approach to modulate the *S* and *σ*. Worthy of extraordinary attention here is the independence between carrier mobility and Ba doping fraction. The substitution of cation sites may generate minimal impact on the valence band as mentioned above, and thus, the carrier mobility is nearly unchanged with doping content [33]. Compared with other high-performance CaMg_2_Bi_2_ materials [33, 42–44], we found that this cation doping will not destroy the inherent high mobility of CaMg_2_Bi_2_ system, which is critical in developing high thermoelectric performance (Figure S1). A rough *μ*_*H*_ ~T^−1.5^ relationship reveals that the primary carrier scattering mechanism is acoustic phonon scattering (Figure 2(b)).

**Figure 3:**
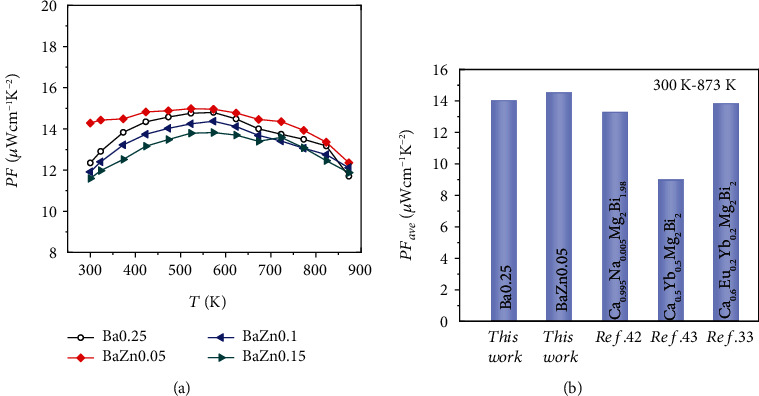
(a) Temperature-dependent *PF* for BaZn*y* (*y* = 0, 0.05, 0.1, 0.15) samples. (b) Comparison of *PF*_*ave*_ for this work with other previous works [33, 42, 43].

Figure 2(c) indicates the temperature-dependent Seebeck coefficient of Ba*x* (*x* = 0, 0.25, 0.5, 0.75) alloys. Clearly, the *S* value increases with increasing Ba fraction, which results from the decreased *n*_*H*_.  Figure 2(d) further shows the calculated Pisarenko relations with *m*^∗^ = 0.63 *m*_*e*_ at room temperature under the assumption of a parabolic band and an acoustic phonon scattering [46]. The better consistency between the fitting line and experiment data demonstrates that the Ba substitution barely disturbs the valence band structure at room temperature. Although Ba-doping evidently improves the *S*, the decay of the *σ* eventually leads to the decline of *PF* (Figure 2(e)). Even so, the *PF* value still remains relatively high in Ba0.25 sample and the *PF*_*ave*_ of 13.9 *μ*W cm^−1^ K^−2^ ranging from 300 K to 873 K is higher than those of other reports [33, 42, 43] (Figure 3(b)).

At the same time, the power factor dependence on carrier concentration at different temperature is plotted in Figure 2(f). The solid lines represent the calculated *PF* on the basis of the assumption that the carrier mobility is independent of *n*_*H*_ (Figure 2(b) and Table S1). It is clear that the peak *PF* can be achieved via adjusting the *n*_*H*_ and the experimental data match well with the calculated *PF*. Particularly, the optimized carrier concentration at room temperature by calculation is around 2.6 × 10^19^ cm^−3^, approximating to the experimental value ~2.5 × 10^19^ cm^−3^ for Ba0.25 sample (Table S1).

**Figure 4:**
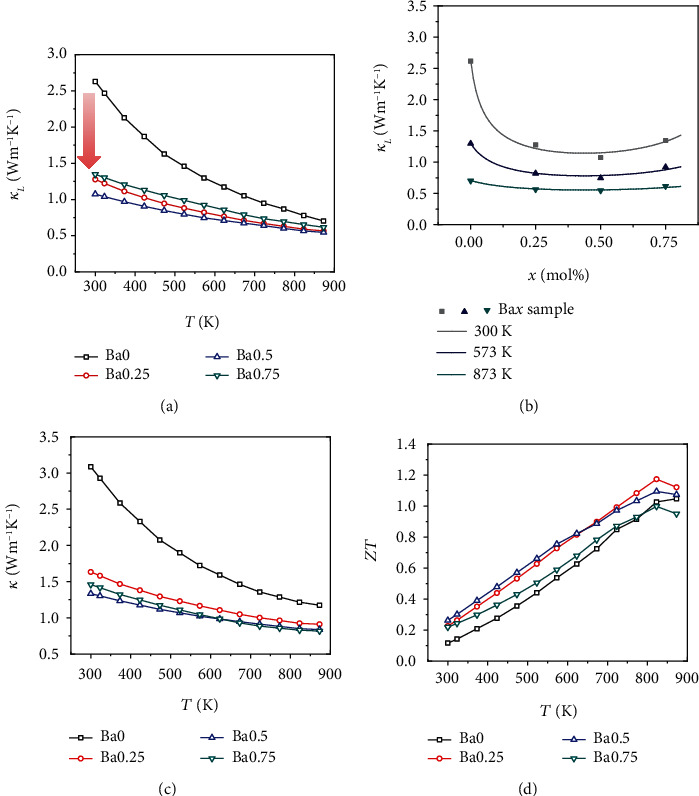
(a) The relationship of *κ*_*L*_ versus temperature for Ba*x* (*x* = 0, 0.25, 0.5, 0.75). (b) Composition dependent lattice thermal conductivity at different temperature with a comparison to model predictions. (c) Temperature dependent thermal conductivity and (d) *ZT* values of Ba*x* samples.

Alloying with Ba could substantially decrease the thermal conductivity as shown in Figure 4. The *κ*_*L*_ could be estimated by subtracting electronic component, *κ*_E_ = *Lσ*T, from the thermal conductivity *κ*, in which the Lorenz factor (*L*) is determined by SPB model assuming the carriers scattering dominated by acoustic phonon. [46] As shown in Figure 4(a), the lattice thermal conductivity first declines with *x*, reaching the minimum value at *x* = 0.5, and then increases. It should be stressed that the room temperature *κ*_*L*_ reaches a lowest value of 1.1 W m^−1^ K^−1^ for Ba0.5 sample, which is 57% lower than that of undoped sample (2.6 W m^−1^ K^−1^). Such low *κ*_*L*_ is mainly due to additional phonon scattering results from point defect caused by the mass and strain field fluctuation between doping atoms (Ba) and host atoms (Ca). Under the hypothesis that the phonon propagation is only restricted by Umklapp process and point defect scattering, the Debye-Callaway model [47, 48] has been used to successfully fit the experimentally measured *κ*_*L*_ in diverse systems, such as YbZn_2_Sb_2-x_Bi_x_ [49], CaZn_2-x_Mg_x_Sb_2_ [50], and CoSbS_1-x_Se_x_ [51]. Thus, to deeply clarify the relationship between point defect and the lattice thermal conductivity, the Debye-Callaway model [47, 48] assuming that the phonon scattering mainly comes from contributions of the Umklapp process and point defect scattering terms is adopted in Ba*x* (*x* = 0, 0.25, 0.5, 0.75) materials. A systematic description about the model can be found in supporting information. The parameter Γ presents the strength of point defect scattering and is regarded as a product of the multiplication of Γ_0_ and *x*_*i*_(1 − *x*_*i*_), where Γ_0_ is a dimensionless parameter obtained utilizing a fitting method and *x*_*i*_ is the fractional concentration. The strength of point defect scattering first increases with increasing Ba fraction until *x* = 0.5 and then decreases (Table S2). The minimal *κ*_*L*_ in the *x* = 0.5 sample mainly attributing to maximal lattice disorder. Furthermore, Figure 4(b) shows a good coincidence between model prediction and measured data, which suggests that Ba/Ca substitution are indeed mainly responsible for the reducing of *κ*_*L*_ in this work.

Combined with the reduced *κ*_*E*_ which is proportional to electrical conductivity, the total thermal conductivity sharply decreases as shown in Figures S2 and 4(c). Specially, the room temperature *κ* decreases from 3.1 W m^−1^ K^−1^ for Ba0 sample to 1.3 W m^−1^ K^−1^ for Ba0.5 sample, with a drop of 58%. Correspondingly, the *κ* at 873 K reduces from 1.2 W m^−1^ K^−1^ to 0.9 W m^−1^ K^−1^, with a decrease of 25%. The *ZT* values as a function of temperature for Ba*x* (*x* = 0, 0.25, 0.5, 0.75) samples are presented in Figure 4(d). Benefiting from the decreased lattice thermal conductivity and appreciable power factor, the maximum *ZT* value of 1.2 at 823 K is obtained for Ba0.25 alloy.

### 2.2. Mg Site Doping and Its Effect on Enhancing TE Properties

Though the *κ*_*L*_ reaches 0.57 W m^−1^ K^−1^ at 873 K for Ba0.25 sample, this value is still much higher than the theoretical limit (0.35 W m^−1^ K^−1^ for Cahill model and 0.14 W m^−1^ K^−1^ for Bvk-Pei model) [44]. How to further decrease *κ*_*L*_ while maintaining the considerable *PF* is another urgent challenge. One of the interesting characteristics of isoelectronic alloying is that they do not bring the charge disorder but introduce the phonon scattering center due to the mass and strain field fluctuations between the host atoms and the guest atoms [52]. In our previous work, we have confirmed firstly that Zn substitution on Mg site can reduce the *κ*_*L*_ without changing the valence band structure [53]. Thus, lower lattice thermal conductivity is prospected in properly BaZn*y* alloys, which is advantageous for enhancing *ZT* values.

Room temperature XRD analysis of BaZn*y* (*y* = 0, 0.05, 0.1, 0.15) samples is displayed in Figure S3. Besides tiny Bi impurity, the main phases of all samples exhibit good agreement with the CaAl_2_Si_2_ structure (space group *P*$\bar{3}$*m*1). It is noted that the lattice parameter increases with Zn content grows up due to the small ionic radius of Zn in comparison with Mg. However, no distinct deviations occur in lattice constant when y exceeds 0.05, which can be attributed to the low solubility limit of Zn in the present compound.

Figure S4 shows temperature-dependent *σ* and *S* for BaZn*y* (*y* = 0, 0.05, 0.1, 0.15). The slightly variation in the electrical conductivity with increasing Zn fraction can be explained by the almost unchanged carrier concentration and mobility (Figure 2(b) and Table 1). Zn doping has a faint effect on the Seebeck coefficient when *y* ≤ 0.05, and the Seebeck coefficients present a downward trend when doping concentration exceed 0.05. The reason for the decline of *S* is still unclear and need further research. One possible explanation is that Zn-related second phase influences the Seebeck coefficient in some degree. As shown in Table 1 and Figure 2(d), the *m*^∗^ is less influenced by doping and all experimental data fall well on the Pisarenko curve, indicating that Zn doping has a tiny influence on the valence band structure at 300 K. In previous literature of the same group [53], it is founded that the carrier mobility of CaMg_1.8_Zn_0.2_Bi_1.98_ decreases with a drop of 20% compared to CaMg_2_Bi_1.98_. However, the carrier mobility is reduced by less than 10% when the Zn doping fraction is less than 0.05. It is generally recognized that the carrier mobility increases with the decrease of carrier concentration. However, in this work, Ba doping reduced carrier concentration, but the carrier mobility remained nearly unchanged rather than increased, further indicating that the alloying scattering caused by Ba doping has a certain effect on the mobility reduction. When we further dope slight Zn (Zn = 0.05) in (Ca_0.75_Ba_0.25_)_0.995_Na_0.005_Mg_2_Bi_1.98_, its weak carrier scattering effect could be obscured by that caused by Ba doping in (Ca_0.75_Ba_0.25_)_0.995_Na_0.005_Mg_2_Bi_1.98_, leading to an inconspicuous change on mobility. Moreover, the experimental carrier concentration of ~2.5 × 10^19^ cm^−3^ for Zn-doped samples is close to the optimal range for maximum PF, as shown in Figure 2(f). The high carrier mobility of ~164 cm^−2^ V^−1^ s^−1^ is still at a higher level, even compared with other doped CaMg_2_Bi_2_ [33, 42–44], as shown in Figure S1.

With increasing Zn content, the *PF* of BaZn*y* samples increases firstly when *y* ≤ 0.05 and then decreases (Figure 3(a)). A conclusion that the appropriate Zn concentration will not deteriorate the electrical transport properties can be made. It is known that the output power density (*ω*) is proportional to the power factor via *ω* = 1/4((*T*_*h*_ − *T*_*c*_)^2^/*L*)*PF* [54]. Thus, the highest *PF*_*ave*_ of 14.4 *μ* W cm^−1^ K^−2^ in BaZn0.05 sample contributes to a higher output power density compared with the other representative reports [33, 42, 43].

**Figure 5:**
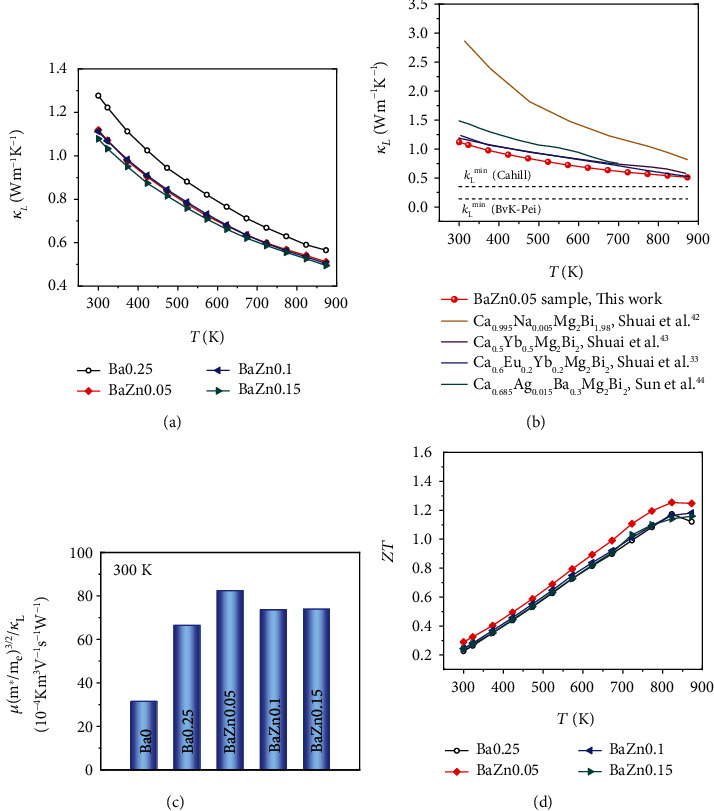
(a) Temperature-dependent the lattice thermal conductivity of BaZn*y* (*y* = 0, 0.05, 0.1, 0.15) samples. (b) Comparison of *κ*_*L*_ between this work and other reported literature [33, 42–44]. The dotted lines represent the theoretical minimum lattice thermal conductivity *κ*_*L*_^min^ (0.35 W m^−1^ K^−1^ for Cahill model and 0.14 W m^−1^ K^−1^ for Bvk-Pei model) [44]. (c) Composition dependent *μ*(m^∗^/me)^3/2^/*κ*_L_ at 300 K for Ba*x* (*x* = 0, 0.25) and BaZn*y* (*y* = 0, 0.05, 0.1, 0.15) samples. (d) The dimensionless figure of merit *ZT* as a function of temperature and content.

Further investigation on thermal transport parameters is carried out. Alloying Zn in Ba0.25 sample indeed effectively reduces the *κ*_*L*_ throughout the measured temperature range due to the enhanced point defects scattering (as shown in Figure 5(a)). The *κ*_*L*_ decreases from ~1.3 W m^−1^ K^−1^ for Ba0.25 sample to 1.1 W m^−1^ K^−1^ BaZn0.05 sample at 300 K and from 0.6 W m^−1^ K^−1^ to 0.5 W m^−1^ K^−1^ at 873 K, which are definitely lower than those of other reported CaMg_2_Bi_2_ systems [33, 42–44] (Figure 5(b)). Specially, the lower *κ*_*L*_ of 0.5 W m^−1^ K^−1^ in this work approaches to the amorphous limit *κ*_*L*_^min^ as shown Figure 5(b) [44], originating from the high concentration point defects caused by Ba and Zn alloying. The reduced lattice thermal conductivity eventually leads to the decline of total thermal conductivity, as shown in Figure S5.

In general, for the intrinsic materials metric, quality factor *β* (*μ*(m^∗^/me)^3/2^/*κ*_L_) was taken as reference to search potential thermoelectric materials [55]. The room temperature *β* of Ba*x*Zn*y* (*x* = 0, 0.25, *y* = 0, 0.05, 0.1, 0.15) is plotted in Figure 5(c). A largest value of 82.4 × 10^−4^ K m^3^ V^−1^ s^−1^ W^−1^ is achieved in the *x* = 0.25, *y* = 0.05 samples, which is about two times higher than that of the Ba0 sample (31.4 × 10^−4^ K m^3^ V^−1^ s^−1^ W^−1^). Thus, with the help of Ba and Zn dual doping, we could realize reduced lattice thermal conductivity without serious compromises in electrical properties. As shown in Figure 5(d), the highest *ZT* value of ~1.25 is achieved for BaZn0.05 sample when it is higher than 823 K.

Snyder and Ursell [56] proposes that the compatibility factor $s=\left(\sqrt{1+ZT}-1\right)/\left(ST\right)$ can be used to facilitate rational materials selection and thermoelectric device design. An optimal relative current density is essential for achieving the maximum conversion efficiency. However, for the segmented power generation modules containing different materials, the electric current flowing in each part should be the same. Thus, the maximum conversion efficiency of each part could be achieved if the compatibility factor of one segment is similar to one another [56]. The situation that a single leg made of a single material is applied within a temperature gradient can be analogous to the different-material segments mentioned above. Each part of such a segment in different temperature may not be capable of achieving the largest conversion efficiency together unless the compatibility factor is independent of temperature. Figure S6 shows that the compatibility factor of Ba and Zn doping are almost temperature independence, which is conducive to maximize efficiency in the whole application temperature range.

**Figure 6:**
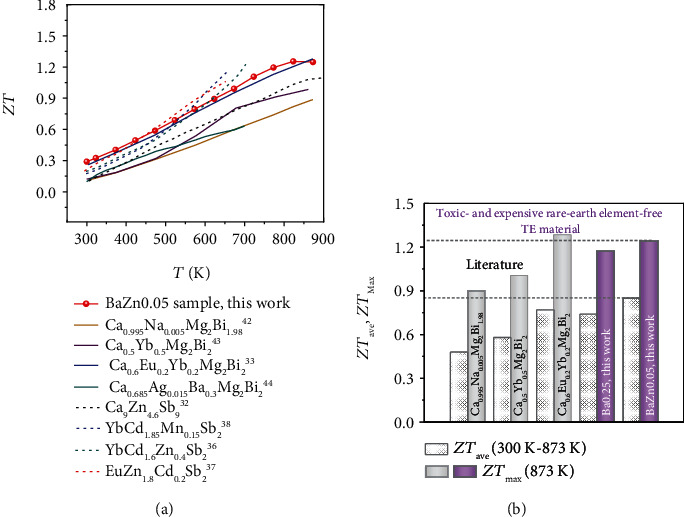
(a) Comparison of *ZT* values between BaZn0.05 sample and other reported samples in p-type AM_2_X_2_ Zintls [32, 33, 36–38, 42–44]. (b) The maximal *ZT*_*max*_ (873 K) and *ZT*_*ave*_ (300 K-873 K) between this work and other previous CaMg_2_Bi_2_ alloys [33, 42, 43].

Compared to other doped CaMg_2_Bi_2_ compounds [33, 42–44], the *ZT* value of 1.25 for BaZn0.05 sample catches up with the current highest level, as shown in Figure 6(a). Besides the appreciable *ZT*, a record *ZT*_*ave*_ of 0.85 is also achieved for the same materials system (Figure 6(b)). The relatively cost-effective, nontoxic, and abundant constituents combined with the high thermoelectric performance makes the present compound more attractive among all thermoelectric material systems.

## 3. Conclusion

In this work, p-type CaMg_2_Bi_2_ was transformed from an uncompetitive thermoelectric material to an outstanding one with record *ZT*_*ave*_ via Ba and Zn dual doping. High-concentration point defects (Ba and Zn doping) play a crucial role in obstructing phonon transport due to the large mass and size difference between the host atom and guest atom. An ultralow *κ*_*L*_ of 0.51 W m^−1^ K^−1^ at 873 K is obtained for (Ca_0.75_Ba_0.25_)_0.995_Na_0.005_Mg_1.95_Zn_0.05_Bi_1.98_ sample. Further, due to the weakly affected carrier mobility, the quality factor *β* will be distinctly strengthened. Eventually, the enhanced *ZT*_*max*_ of 1.25 at 873 K and the record *ZT*_*ave*_ of 0.85 between 300 K and 873 K are also obtained. Last but not least, just this material also displays the temperature independent compatibility factor. Taking into account the practical design of thermoelectric device, high-performance thermoelectric materials composed of cost-effective, environmentally friendly, and plentiful elements are of greatly practical significance for large-scale commercial applications.

## Figures and Tables

**Table 1:** Room temperature electrical transport parameters of BaZn*y* (*y* = 0, 0.05, 0.1, 0.15) samples.

Sample number	Composition	*n*_*H*_ (10^19^ cm^−3^)	*μ*_*H*_ (cm^−2^V^−1^s^−1^)	*m* ^∗^ (m_e_)	*σ* (10^3^ Sm^−1^)	*S* (*μ*VK^−1^)
Ba0.25	(Ca_0.75_Ba_0.25_)_0.995_Na_0.005_Mg_2_Bi_1.98_	2.5	169	0.63	66.8	136
BaZn0.05	(Ca_0.75_Ba_0.25_)_0.995_Na_0.005_Mg_1.95_Zn_0.05_Bi_1.98_	2.5	170	0.66	68.6	144
BaZn0.1	(Ca_0.75_Ba_0.25_)_0.995_Na_0.005_Mg1.9Zn0.1Bi_1.98_	2.5	166	0.62	65.3	135
BaZn0.15	(Ca_0.75_Ba_0.25_)_0.995_Na_0.005_Mg1.85Zn0.15Bi_1.98_	2.5	168	0.61	68.4	130

## Supplementary Materials

Supplementary Materials Experimental Section: sample preparation and sample characterization. Figure S1: carrier mobility *vs* carrier concentration between this work and other literature of CaMg_2_Bi_2_ materials. Figure S2: temperature-dependent the electronic thermal conductivity of Ba*x* (*x* = 0, 0.25, 0.5, 0.75) samples. Figure S3: (a) the XRD patterns of BaZn*y* (*y* = 0, 0.05, 0.1, 0.15). (b) Enlarged view of XRD patterns between 35° and 39°. (c) Lattice constant as a function of composition. Figure S4: temperature-dependent (a) the electrical conductivity and (b) the Seebeck coefficient of BaZn*y* (*y* = 0, 0.05, 0.1, 0.15). Figure S5: the thermal conductivity as a function of temperature for BaZn*y* (*y* = 0, 0.05, 0.1, 0.15). Figure S6: the compatibility factors *vs* temperature for Ba*x* (*x* = 0, 0.25) and BaZn*y* (*y* = 0.05, 0.1, 0.15) sample. Table S1: room temperature electrical transport parameters of Ba*x* (*x* = 0, 0.25, 0.5, 0.75). Calculation of lattice thermal conductivity using the Callaway Model.
