# Supplementary material for: Achieving High Thermoelectric Performance in Rare-Earth Element-Free CaMg2Bi2 with High Carrier Mobility and Ultralow Lattice Thermal Conductivity
Source: Research (Wash D C). 2020 Jul 24;2020:5016564. doi: 10.34133/2020/5016564 (PMC7396126; doi:10.34133/2020/5016564)
Supplement: Supplementary Materials — Experimental Section: sample preparation and sample characterization. Figure S1: carrier mobility vs carrier concentration between this work and other literature of CaMg2Bi2 materials. Figure S2: temperature-dependent the electronic thermal conductivity of Bax (x = 0, 0.25, 0.5, 0.75) samples. Figure S3: (a) the XRD patterns of BaZny (y = 0, 0.05, 0.1, 0.15). (b) Enlarged view of XRD patterns between 35° and 39°. (c) Lattice constant as a function of composition. Figure S4: temperature-dependent (a) the electrical conductivity and (b) the Seebeck coefficient of BaZny (y = 0, 0.05, 0.1, 0.15). Figure S5: the thermal conductivity as a function of temperature for BaZny (y = 0, 0.05, 0.1, 0.15). Figure S6: the compatibility factors vs temperature for Bax (x = 0, 0.25) and BaZny (y = 0.05, 0.1, 0.15) sample. Table S1: room temperature electrical transport parameters of Bax (x = 0, 0.25, 0.5, 0.75). Calculation of lattice thermal conductivity using the Callaway Model. [file 5016564.f1.docx]

**Supporting Information**

**Achieving** **High Thermoelectric Performance in Rare-earth Element-free CaMg_2_Bi_2_ with High Carrier Mobility and Ultralow Lattice Thermal Conductivity**

Muchun Guo^a^‡, Fengkai Guo^a^‡, Jianbo Zhu^a^, Li Yin^b^, Qian Zhang^b^, Wei Cai^a^, and Jiehe Sui^a*^

*^a^National Key Laboratory for Precision Hot Processing of Metals, Harbin Institute of Technology Harbin 150001, China*

*^b^Department of Materials Science and Engineering, Harbin Institute of Technology*

*Shenzhen, Guangdong 518055, China*

‡*Equal contributors*

*To whom correspondence should be addressed. E-mail: [suijiehe@hit.edu.cn](mailto:suijiehe@hit.edu.cn)

**Experimental Section**

**Sample preparation**

The raw elements with high purity (> 99.9%) were weighted according to the nominal composition of (Ca_1-x_Ba_x_)_0.995_Na_0.005_Mg_2_Bi_1.98_ (*x* = 0, 0.25, 0.5 ,0.75) and (Ca_0.75_Ba_0.25_)_0.995_Na_0.005_Mg_2-_*_y_*Zn*_y_*Bi_1.98_ (*y* = 0, 0.05, 0.1, 0.15) inside an Ar protected glove box. In order to simplify the expression, the samples of (Ca_1-x_Ba_x_)_0.995_Na_0.005_Mg_2_Bi_1.98_ are named as Ba*x* (*x* = 0, 0.25, 0.5 ,0.75) alloys and the Zn doped samples with Ba content of 0.25, (Ca_0.75_Ba_0.25_)_0.995_Na_0.005_Mg_2-_*_y_*Zn*_y_*Bi_1.98_ (*y* = 0, 0.05, 0.1, 0.15), are simplified as BaZn*y* alloys, i.e., Ba0, Ba0.25, Ba0.5, Ba0.75 and BaZn0.05, BaZn0.1, BaZn0.15 samples. The weighted elements were sealed in stainless-steel jar under Ar atmosphere and mechanically alloyed in a high-energy ball mill (SPEX 8000M) for 12 h. Then, the ball-milled powders were loaded into a graphite die with a diameter of 12.7 mm and quickly compacted using spark plasma sintering (SPS) at 923 K with an axial pressure of 60 MPa for 5 min.

**Sample Characterization**

The phase composition of dense pellets was analyzed by X-ray diffraction (XRD) with Cu Kα (λ = 1.5406 Å) radiation. The simultaneous measurement of electrical conductivity and Seebeck coefficient were implemented on a commercial ZEM-3 (ULVAC, Japan) instrument from 300 K to 873 K. The total thermal conductivity was calculated using the relationship *κ* = *D* × *C*_p_ × *d*, where *D* is thermal diffusivity measured on the laser flash apparatus (Netzsch LFA 427), *C*_p_ is specific heat determined by the Dulong-Petit approximation and *d* is the density obtained via Archimedes method. In addition, the Hall carrier concentration *n*_H_ and mobility *μ*_H_ were calculated by *n*_H_ = 1/(e*R*_H_*)* and *μ*_H_ = *σ*/(e*n*_H_), where *R*_H_ is Hall coefficient acquired by using the van der Pauw technique under a magnetic field of 1.5 T, *e* is the electronic charge and the σ is the electrical conductivity.

**Figure S1.** Carrier mobility vs carrier concentration between this work and other literature of CaMg_2_Bi_2_ materials [1-4].

**Figure S2.** Temperature-dependent the electronic thermal conductivity of Ba*x* (*x* = 0, 0.25, 0.5 ,0.75) samples.


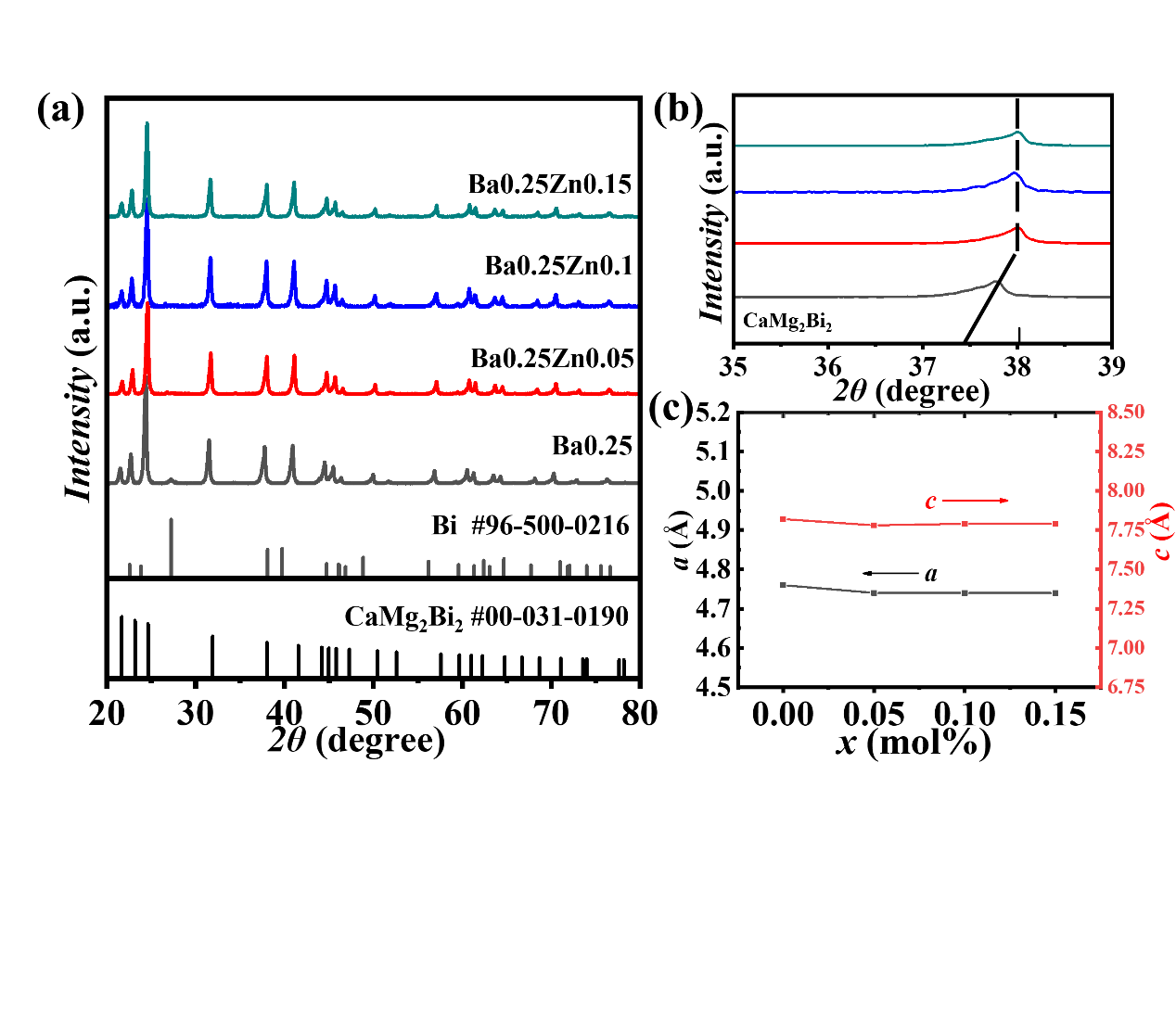


**Figure S3.** (a) The XRD patterns of BaZn*y* (*y* = 0, 0.05, 0.1, 0.15). (b) Enlarged view of XRD patterns between 35° and 39°. (c) Lattice constant as a function of composition.


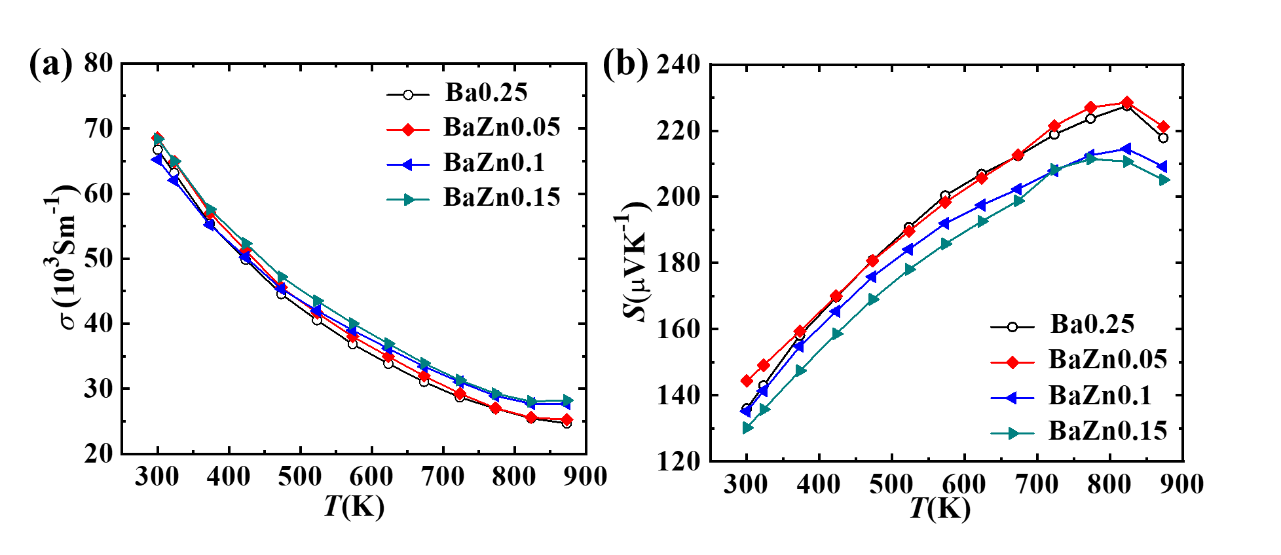


**Figure S4.** Temperature dependent (a) the electrical conductivity and (b) the Seebeck coefficient of BaZn*y* (*y* = 0, 0.05, 0.1, 0.15).

**Figure S5.** The thermal conductivity as a function of temperature for BaZn*y* (*y* = 0, 0.05, 0.1, 0.15).

**Figure S6.** The compatibility factors *vs* temperature for Ba*x* (*x* = 0, 0.25) and BaZn*y* (*y* = 0.05, 0.1, 0.15) sample.

**Table S1** **Room temperature electrical transport parameters of** Ba*x* (*x* = 0, 0.25, 0.5, 0.75)**.**

| Sample  Number | | Composition | *n*_H_  (10^19^ cm^-3^) | | | μ_H_  (cm^-2^V^-1^s^-1^) | m*  (m_e_) | *σ*  (10^3^ Sm^-1^) | *S*  (μVK^-1^) |
| --- | --- | --- | --- | --- | --- | --- | --- | --- | --- |
| Ba0 | Ca_0.995_Na_0.005_Mg_2_Bi_1.98_ | | | 3.2 | 160 | | 0.64 | 82.9 | 120.5 |
| Ba0.25 | (Ca_0.75_Ba_0.25_)_0.995_Na_0.005_Mg_2_Bi_1.98_ | | | 2.5 | 169 | | 0.63 | 66.8 | 136.0 |
| Ba0.5 | (Ca_0.5_Ba_0.5_)_0.995_Na_0.005_Mg_2_Bi_1.98_ | | | 2.0 | 159 | | 0.61 | 50.2 | 151.7 |
| Ba0.75 | (Ca_0.25_Ba_0.75_)_0.995_Na_0.005_Mg_2_Bi_1.98_ | | | 0.9 | 167 | | 0.64 | 23.2 | 214.0 |

***Calculation of lattice thermal conductivity using the Callaway Model***

In this work, the Debye-Callaway model is employed in Ba*x* samples for further comprehend the effect of point defects on reducing *κ*_L_ when Umklapp process and point defect scattering are dominant [5, 6].

$\frac{\kappa_{L}}{\kappa_{L}^{p}}$ =$\frac{{tan}^{-1}(u)}{u}$ (S1)

*u^2^* = $\frac{\pi^{2}\theta_{D}\Omega}{hv^{2}}$ $\kappa_{L}^{p}\Gamma$ (S2)

where$\kappa_{L}^{p}$ is pure *κ*_L_ of Ba0 sample, *u* is scaling parameter, *h* is Planck constant. We use the known θ_D_ for CaMg_2_Bi_2_, namely θ_D_ = 248 K [1]. The mean sound speed (*υ*) of 2581 m/s is obtained based on the relationship of $\theta_{D}$ = $\frac{h}{\kappa_{B}}$[$\frac{3N}{4\pi V}$]^1/3^*υ*, in which *N* is the number of atoms in a unit cell and *V* is the unit-cell volume. *N* = 5 and *V* = 0.1488 nm^3^ for CaMg_2_Bi_2_ [7]. Ω is the average volume per atom. The experimental scaling parameter *Γ* can be split into the mass ($\frac{{\Delta M}_{i}}{M}$) and strain ($\frac{{\Delta\delta}_{i}}{\delta}$) parts, respectively. The formula is written as follow:

$\Gamma= \Sigma_{i}x_{i}$(1-$x_{i}$)[${(\frac{{\Delta M}_{i}}{M})}^{2}$+$\varepsilon{(\frac{{\Delta\delta}_{i}}{\delta})}^{2}$] (S3)

Where *x_i_* is the fractional concentration, *M* is average atomic mass and δ is assumed to the lattice parameter of intrinsic materials. In addition, *ε* is a phenomenological parameter and difficult to calculate accurately because there are usually micro stresses and other defects in practical materials. Thus, we regard *Γ* as a product of the multiplication of *Γ_0_* and *x_i_*(1-*x_i_*):

*Γ* = *Γ_0_* × *x_i_*(1-*x_i_*) (S4)

where *Γ_0_* is a dimensionless parameter obtained via utilize a fitting method. Compared with the large number of calculations, the fitting method is simple and can reflect the effect of point defects well.

**Table S2** **Lattice thermal conductivity coefficients simulated by Eq S1-S4.**

| Parameter | 300 K  *Γ_0_* = 12.1043 | | 573 K  *Γ_0_* = 9.57635 | 873 K  *Γ_0_* = 8.44486 |
| --- | --- | --- | --- | --- |
|  | *x_i_*(1-*x_i_*) | *Γ_300 K_* | *Γ_573 K_* | *Γ_873 K_* |
| *x* = 0 | 0 | 0 | 0 | 0 |
| *x* = 0.25 | 0.1875 | 2.27 | 1.80 | 1.58 |
| *x* = 0.5 | 0.25 | 3.03 | 2.39 | 2.11 |
| *x* = 0.75 | 0.1875 | 2.27 | 1.8 | 1.58 |

Reference

1. J. Shuai, H.Y. Geng, Lan et al., "Higher thermoelectric performance of Zintl phases (Eu_0.5_Yb_0.5_)_1-x_Ca_x_Mg_2_Bi_2_ by band engineering and strain fluctuation," *Proceedings of the National Academy of Sciences of the United States of America*, vol. 113, no. 29, pp. E4125-E4132, 2016.

2. C. Sun, X.M. Shi, Zheng et al., "Transport properties of p-type CaMg_2_Bi_2_ thermoelectrics," *Journal of Materiomics*, vol.5, no.4, pp. 567-573, 2019.

3. J. Shuai, H.S. Kim, Liu et al., "Thermoelectric properties of Zintl compound Ca_1−x_Na_x_Mg_2_Bi_1.98_," *Applied Physics Letters*, vol. 108, no, 18, ariticle 183901, 2016.

4. J. Shuai, Z.H. Liu, H.S. Kim et al., "Thermoelectric properties of Bi-based Zintl compounds Ca_1−x_Yb_x_Mg_2_Bi_2_." *Journal of Materials Chemistry A*, vol. 4, no.11, pp. 4312-4320, 2016.

5. J. Callaway, H.C. von Baeyer, "Effect of Point Imperfections on Lattice Thermal Conductivity," *Physical Review*, vol. 120, no. 4, pp.1149-1154, 1960.

6. P.G. Klemens, "Thermal Resistance due to Point Defects at High Temperatures," *Physical Review*, vol. 119, no. 2, pp. 507-509, 1960.

7. A.F. May, M.A. McGuire, D.J. Singh et al., "Structure and properties of single crystalline CaMg_2_B_i2_, EuMg_2_Bi_2_, and YbMg_2_Bi_2_," *Inorganic Chemistry*, vol. 50 no. 21, pp. 11127-11133, 2011.
